# Trends in Asthma‐Rhinitis Allergic Multimorbidity and Polysensitization in China: The CARRAD Study

**DOI:** 10.1002/mco2.70762

**Published:** 2026-06-04

**Authors:** Wanjun Wang, Jianhong Wang, Guihua Song, Hua Xie, Rongfei Zhu, Yong He, Jun Tang, Junge Wang, Jinghua Yang, Lili Zhi, Lin Wu, Yan Jiang, Xiaoqin Zhou, Dongming Huang, Ning Wang, Rui Xu, Yuan Gao, Zhimin Chen, Xiaoli Han, Guolin Tan, Jinzhun Wu, Deyu Zhao, Jianjun Chen, Weixi Zhang, Yuemei Sun, Yi Jiang, Weitian Zhang, Qianhui Qiu, Chuanhe Liu, Jie Yin, Guodong Hao, Huabin Li, Yongsheng Xu, Shaohua Chen, Shi Chen, Juan Meng, Dan Zeng, Wei Tang, Chuangli Hao, Nanshan Zhong, Jing Li, the China Alliance of Research on Respiratory Allergic Disease (CARRAD)

**Affiliations:** ^1^ Department of Allergy and Clinical Immunology National Clinical Research Center for Respiratory Disease State Key Laboratory of Respiratory Disease Guangzhou Institute of Respiratory Health The First Affiliated Hospital of Guangzhou Medical University Guangzhou China; ^2^ The First People's Hospital of Yibin Yibin China; ^3^ The First Affiliated Hospital of Henan University of Traditional Chinese Medicine Zhengzhou Henan China; ^4^ General Hospital of Northern Theater Command Shenyang China; ^5^ Tongji Hospital Tongji Medical College Huazhong University of Science & Technology Wuhan China; ^6^ The Affiliated Hospital of Medical School Ningbo University Ningbo China; ^7^ Foshan First People's Hospital Foshan China; ^8^ Beijing Hospital of Traditional Chinese Medicine Beijing China; ^9^ Guangdong Provincial Hospital of Chinese Medicine Guangzhou China; ^10^ The First Affiliated Hospital of Shandong First Medical University Shandong Institute of Respiratory Diseases Taian China; ^11^ Hangzhou Hospital of Traditional Chinese Medicine Hangzhou China; ^12^ The Affiliated Hospital of Qingdao University Qingdao China; ^13^ Hubei Province Maternal and Child Health Hospital Hubei China; ^14^ Boai Hospital of Zhongshan City Zhongshan China; ^15^ Xi'an Children's Hospital Xi'an China; ^16^ The First Affiliated Hospital of Sun Yat‐Sen University Guangzhou China; ^17^ The First Affiliated Hospital of Zhengzhou University Zhengzhou China; ^18^ Children's Hospital of Zhejiang University School of Medicine National Clinical Research Center for Child Health Hangzhou China; ^19^ Hebei General Hospital Hebei China; ^20^ Third Xiangya Hospital of Central South University Changsha China; ^21^ The Women and Children's Hospital Affiliated to Xiamen University Xiamen China; ^22^ Children's Hospital of Nanjing Medical University Nanjing China; ^23^ Union Hospital of Tongji Medical College Wuhan China; ^24^ The Second Affiliated Hospital and Yuying Children's Hospital of Wenzhou Medical University Wenzhou China; ^25^ Yu Huang Ding Hospital Yantai China; ^26^ The First Hospital of Shanxi Medical University Taiyuan China; ^27^ Shanghai Jiao Tong University Affiliated Sixth People's Hospital Shanghai China; ^28^ Zhujiang Hospital of Southern Medical University Guangzhou China; ^29^ Children's Hospital Capital Institute of Pediatrics Beijing China; ^30^ Chengdu First People's Hospital Chengdu China; ^31^ Tangshan Gongren Hospital Tangshan China; ^32^ ENT Institute and Department of Otorhinolaryngology Eye & ENT Hospital Fudan University Shanghai China; ^33^ Children's Hospital of Tianjin University Tianjin China; ^34^ Guangdong Provincial People's Hospital Guangzhou China; ^35^ Hainan Provincial People's Hospital Haikou China; ^36^ West China Hospital of Sichuan University Chengdu China; ^37^ Chongqing General Hospital University of Chinese Academy of Sciences Chongqing China; ^38^ Ruijin Hospital of Shanghai Jiaotong University Shanghai China; ^39^ Children's Hospital of Soochow University Suzhou China

**Keywords:** allergic comorbidities, allergic rhinitis, asthma, epidemiology, polysensitization

## Abstract

The trends in allergic comorbidities secondary to the environmental variations in China remain unclear. We aimed to determine the variation of allergic comorbidities and polysensitization among asthma and/or rhinitis patients in the past decade. We assessed two nationally representative cross‐sectional datasets from 2008 to 2009 and 2018 to 2019, which enrolled 2322 and 2353 patients, respectively. Over the present 10‐year study period, the prevalence of allergic symptoms and allergen sensitivity among patients with multiple sensitivities in the 2018–2019 cohort was significantly higher than that in the 2008–2009 cohort, especially for mites, pollen, and animal allergens. The comorbidity rates of asthma, allergic rhinitis, conjunctivitis, and eczema were significantly increased in the 2018–2019 cohort. Also in that cohort, IgE polysensitization was significantly associated with the coexistence of asthma and rhinitis, and the number of IgE‐reactive allergens was significantly associated with the number of multimorbidities. Use of an air‐conditioner and carpet in the home, and keeping pet were linked to the risk of polysensitization. Our findings suggest an increase in the comorbidity rate and multimorbid polysensitized phenotype of allergic diseases in China. Asthma occurred in both cohorts more frequently with coexisting allergies than as a single entity.

## Graphical abstract

This cross‐sectional study highlights the increasing prevalence of allergic diseases and comorbidities under environmental conditions of rapid modernization in China. Our observations underline the relevance of the allergic comorbidities phenotype at the clinical and public health levels.

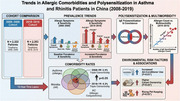

## 1 Introduction

The global prevalence of allergic diseases is closely linked to the social and environmental changes. In China, one of the most rapidly urbanizing countries globally, the China Allergy and Respiratory Research Alliance (CARRAD) conducted two nationwide epidemiologic surveys spanning 10 years, namely, CARRAD‐I (2008–2009) and CARRAD‐II (2018–2019). Preliminary studies indicate that changes in climate, vegetation, and indoor environments have led to alterations in the prevalence and risk factors of allergic diseases in China [1, 2]. These factors are believed to have jointly promoted the complexity of allergic reactions via epigenetic regulation and immune imbalance mechanisms [3]. However, existing research often focuses on the cross‐sectional epidemiologic characteristics of a single instance [4]. Systematic evidence remains limited regarding the dynamic evolution of multiple allergic comorbidities and polysensitization, especially variations over a long period, driven by environmental factors.

Research has revealed that the phenomenon of allergic comorbidities may be associated with acceleration of the “allergic march,” which is the process via which individuals gradually progress from atopic dermatitis in infancy to allergic rhinitis (AR), asthma, and other multisystem diseases [5, 6]. Immunoglobulin E (IgE)‐mediated multiple sensitization (defined as sensitivity to ≥3 types of allergen) is confirmed to be associated with severe clinical symptoms and treatment resistance [7]. However, the evolution of this phenotype in the context of rapid modernization and its association with comorbidities remain unclear. In the present study, we investigate the following issues: (1) whether there has been a significant change in multiple sensitization among Chinese patients with asthma and/or rhinitis over the past decade; (2) whether there is a relationship between multiple IgE sensitization and the incidence of allergic comorbidities. Using data obtained from standardized questionnaires, serum‐specific IgE (sIgE) testing, and multivariate logistic regression models, we systematically evaluated the 10‐year trajectory of the transition of allergic diseases from monosensitization to polysensitization.

Tremendous transformations have taken place in China such as more westernized living and working styles, fast modernization, and increases in temperature and humidity. These have been identified as the significant factors, which may contribute to the rising incidence of allergen sensitization [1, 2]. This analysis of cross‐sectional epidemiologic data highlights the increasing prevalence of allergic diseases comorbidity under current conditions of modernization in China.

Our findings also suggest that the mechanisms involved in the association between IgE polysensitization and asthma phenotypes are partly enhanced. Our observations underline the relevance of this phenotype at the clinical and public health levels.

## 2 Results

### 2.1 Comparison of Monosensitized and Polysensitized Participants in the Two Cohorts

Table 1 summarizes the demographics and clinical traits of the two cohorts. The prevalence of polysensitization was similar between men and women. There were significantly more monosensitized patients with asthma in the 2018–2019 cohort than in the 2008–2009 cohort (48.8% vs. 40.1%, *p* < 0.001). Monosensitized patients in the 2018–2019 cohort had a higher percentage of nose, eye, and skin symptoms than did those in the 2008–2009 cohort. The proportion with a family atopic history was higher in the polysensitized group than that in the monosensitized group in both the 2008–2009 and 2018–2019 cohorts. Moreover, monosensitized patients in the 2018–2019 cohort had higher rates of mite, pollen, and animal allergy than did those in the 2008–2009 cohort. A significantly greater proportion of polysensitized participants had asthma, AR, and conjunctivitis in the 2018–2019 cohort than did those in the 2008–2009 cohort (60.4% vs. 55.3%, 86.8% vs. 80.6%, 52.8% vs. 48.1%, 29.5% vs. 23.4%, respectively; *p* < 0.05). The percentage of participants with physical symptoms was higher in the 2018–2019 cohort than that in the 2008–2009 cohort. Mite, pollen, and fungus allergies were more frequent among patients in the 2018–2019 cohort than among those in the 2008–2009 cohort.

**TABLE 1:** Comparison of characteristics between monosensitized and polysensitized patients in China.

	Monosensitized patients					Polysensitized patients				
Study year	2008–2009, *N* = 1058		2018–2019, *N* = 929		*p* value	2008–2009, *N* = 1264		2018–2019, *N* = 1424		*p* value
Sex, *n* (%)										
Male	592	(56.0)	510	(54.9)	0.636	680	(53.8)	763	(53.6)	0.942
Female	466	(44.0)	419	(45.1)		584	(46.2)	661	(46.4)	
Age (years), median (IQR)	22.0 (8.0; 37.0)		23.0 (9.0; 35.0)		0.783	21.0 (9.0; 36.0)		22.0 (13.0; 39.0)		0.515
Diagnosis, *n* (%)										
AS	424	(40.1)	453	(48.8)	**< 0.001**	699	(55.3)‡	860	(60.4)‡	**0.008**
AR	692	(65.4)	634	(68.2)	0.180	1019	(80.6)‡	1236	(86.8)‡	**< 0.001**
AC	386	(36.5)	362	(39.0)	0.254	608	(48.1)‡	752	(52.8)‡	**0.015**
UC/eczema	240	(22.7)	190	(20.4)	0.228	296	(23.4)	420	(29.5)‡	**< 0.001**
Anaphylaxis	20	(1.9)	11	(1.2)	0.205	29	(2.3)	51	(3.6)‡	0.056
Family history of atopy, *n* (%)	530	(50.1)	427	(46.0)	0.129	742	(58.7)‡	968	(68.0)‡	**< 0.001**
Physical symptoms, *n* (%)										
Seasonal	290	(27.4)	290	(31.2)	0.134	306	(24.2)	550	(38.6)‡	**< 0.001**
Perennial	399	(37.7)	379	(40.8)	0.263	576	(45.6)‡	815	(57.2)‡	**< 0.001**
Nose symptoms	765	(72.3)	746	(80.3)	**0.001**	956	(75.6)	1225	(86.0)‡	**< 0.001**
Itchy, watery eyes	442	(41.8)	454	(48.9)	**0.009**	611	(48.3)^a^	786	(55.2)‡	**0.003**
Itchy rash	227	(21.5)	303	(32.6)	**< 0.001**	317	(25.1)^a^	564	(39.6)‡	**< 0.001**
Attacks of wheezing	571	(54.0)	527	(56.7)	0.327	789	(62.4)‡	953	(66.9)‡	**0.042**
Allergen sIgE positivity, *n* (%)										
Any mites	586	(55.4)	579	(62.3)	**0.002**	853	(67.5)‡	1182	(83.0)‡	**< 0.001**
Any pollen	156	(14.7)	220	(23.7)	**< 0.001**	416	(32.9)‡	552	(38.8)‡	**0.002**
Any animal dander	53	(5.0)	85	(9.2)	**< 0.001**	236	(18.7)‡	298	(20.9)‡	0.143
Any mold	161	(15.2)	133	(14.3)	0.573	211	(16.7)	310	(21.8)‡	**0.001**
Lifestyle factors										
Living room and bedroom A/C use	345	(32.6)	857	(92.3)	**< 0.001**	482	(38.1)	1346	(94.5)	**< 0.001**
Bedroom A/C use	672	(63.5)	759	(81.7)	**< 0.001**	974	(77.1)	1302	(91.4)	**< 0.001**
Mattress use	873	(82.5)	812	(87.4)	**0.002**	1078	(85.3)	1292	(90.7)	**< 0.001**
Owning pets	139	(13.1)	136	(14.6)	0.334	190	(15)	248	(17.4)	0.095
Household floor material										
Wood	176	(16.6)	93	(10)	**< 0.001**	231	(18.3)	169	(11.9)	**< 0.001**
Carpet	152	(14.4)	211	(22.7)	**< 0.001**	214	(16.9)	121	(8.5)	**< 0.001**
Bedroom floor material										
Wood	183	(17.3)	164	(17.6)	0.835	241	(19.1)	244	(17.1)	0.194
Carpet	227	(21.5)	269	(29)	**0.001**	225	(17.8)	473	(33.2)	**< 0.001**
Diet (two or more times per week)										
Red meat	212	(20)	323	(34.8)	**< 0.001**	231	(18.3)	443	(31.1)	**< 0.001**
Fried fish	314	(29.7)	101	(10.9)	**< 0.001**	475	(37.6)	232	(16.3)	**< 0.001**
Fresh fruits	226	(21.4)	329	(35.4)	**< 0.001**	329	(26)	553	(38.8)	**< 0.001**
Raw vegetables	113	(10.7)	169	(18.2)	**< 0.001**	139	(11)	251	(17.6)	**< 0.001**
Fruit juices	141	(13.3)	63	(6.8)	**< 0.001**	153	(12.1)	100	(7)	**< 0.001**
Soft drinks	87	(8.2)	48	(5.2)	**0.007**	119	(9.4)	81	(5.7)	**< 0.001**

Abbreviations: A/C, air‐conditioner; AC, allergic conjunctivitis; AR, allergic rhinitis; AS, asthma; IQR, interquartile range; sIgE, specific immunoglobulin E; UC, urticaria.

^a^ *p* < 0.05, ‡*p* < 0.001 compared with monosensitized patients in the 2008–2009 or 2018–2019 cohort.

Bold text indicates statistical significance (*p* < 0.05). Percentages may not total 100 owing to rounding.

### 2.2 Prevalence of Allergic Comorbidities in the Two Cohorts

**FIGURE 1:**
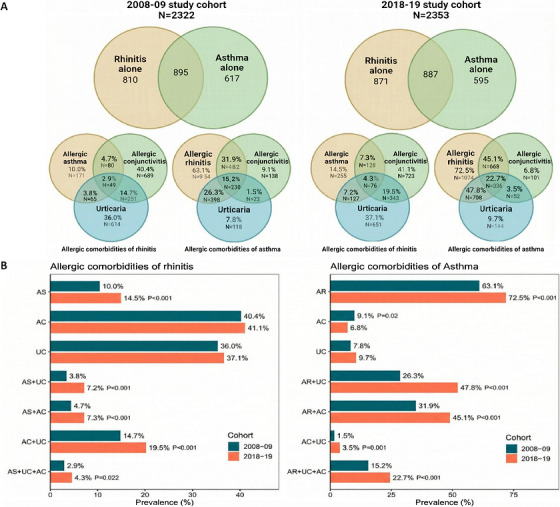
Prevalence of allergic comorbidities in the two cohorts. AC, allergic conjunctivitis; AR, allergic rhinitis; AS, asthma; UC, urticaria.

Figure 1 Venn diagrams illustrates the associations among the frequency for allergic asthma, AR, allergic conjunctivitis (AC), and urticaria. The percentage of patients with rhinitis combined with allergic asthma, AC, or UC in the 2008–2009 cohort was 10.0%, 40.4%, and 36.0%, respectively. These rates were higher in the 2018–2019 cohort, with a prevalence of 14.5%, 41.1%, and 37.1%. The prevalence of asthma with comorbid AR, AC, or UC in the 2008–2009 cohort was 63.1%, 9.1%, and 7.8%. The prevalence of asthma combined with AR was highest in the 2018–2019 cohort at 72.5%. The prevalence of comorbid allergic asthma, AC, and UC in rhinitis was 2.9% in the 2008–2009 cohort; this rate was higher in the 2018–2019 cohort at 4.3%. The prevalence of comorbid AR, AC, and UC in asthma was 15.2% in the 2008–2009 cohort, and this rate was higher in the 2018–2019 cohort at 22.7%.

### 2.3 Allergen Profile With Allergic Comorbidity Among Patients With Asthma in the Two Cohorts

Table 2 details the allergen profile with allergic comorbidity of asthmatic patients in two cohorts. The respective prevalence values of mite allergy to *D. pteronyssinus* and *D. farinae* among patients with asthma and AR (64.3%, 62.9%), AC (54.4%, 53.2%), UC (52.5%, 51.9%), AR + AC (68%, 65.1%), or AR + UC (63.7%, 61.4%) were all higher in the 2018–2019 cohort than those in the 2008–2009 cohort. Only the prevalence of cat allergy in asthma with AR + UC (10.8%) was lower in the 2018–2019 cohort, as compared with that in the 2008–2009 cohort (15.9%). Among patients with asthma and AR + UC, the prevalence of allergy to timothy grass (9.7%) and *A. vulgaris* (14.9%) was higher in the 2018–2019 cohort. In patients with asthma and AR + AC + UC, the proportions with mite allergy to *D. pteronyssinus* (68.2%), *D, farinae* (66.5%), and *B. tropicalis* (40.1%), as well as pollen allergy to *A. artemisiifolia* (13.8%) and *A. vulgaris* (20.3%) were all higher in the 2018–2019 cohort than those in the 2008–2009 cohort.

**TABLE 2:** Prevalence of allergen sensitization in allergic comorbidity among patients with asthma.

Allergic comorbidity of asthma	AR		AC		UC		AR + AC		AR + UC		AC + UC		AR + AC + UC	
Study year	2008–2009, *N* = 954	2018–2019, *N* = 1074	2009–2009, *N* = 138	2018–2019, *N* = 101	2009–2009, *N* = 118	2018–2019, *N* = 144	2009–2009, *N* = 482	2018–2019, *N* = 668	2009–2009, *N* = 398	2018–2019, *N* = 708	2009–2009, *N* = 23	2018–2019, *N* = 52	2009–2009, *N* = 230	2018–2019, *N* = 336
*Dermatophagoides pteronyssinus*	387 (40.6)	691 (64.3)‡	53 (38.6)	55 (54.4)†	49 (41.4)	76 (52.5)†	206 (42.8)	454 (68.0)‡	157 (39.5)	451 (63.7)‡	10 (41.4)	34 (65.0)	106 (46.0)	229 (68.2)‡β′γ′
*Dermatophagoides farinae*	370 (38.8)	676 (62.9)‡	51 (36.6)	54 (53.2)†	47 (39.6)	75 (51.9)†	201 (41.7)	435 (65.1)‡	153 (38.4)	435 (61.4)‡	9 (39.6)	32 (62.3)	102 (44.2)	223 (66.5)‡β′γ′
*Blomia tropicalis*	281 (29.5)	**423 (39.4)‡**	35 (25.2)	33 (32.5)	25 (20.9)	46 (32.1)†	138 (28.6)	278 (41.6)‡	89(22.4)α′ω	274 (38.7)‡	6 (27.8)	20 (37.8)	64 (28.0)	135 (40.1)‡
Cat dander	130 (13.6)	141 (13.1)	19 (13.9)	11 (10.9)	17 (14.3)	13 (8.9)	71 (14.8)	83 (12.4)	63 (15.9)	76 (10.8)†	3 (13.1)	7 (12.5)	22 (9.6)	39 (11.7)
Dog dander	62 (6.5)	89 (8.3)	9 (6.6)	7 (6.8)	6 (5.1)	9 (6.5)	26 (5.3)	50 (7.5)	29 (7.4)	52 (7.3)	2 (6.6)	5 (8.8)	17 (7.5)	29 (8.7)
Timothy grass	44 (4.6)	57 (5.3)	7 (5.2)	6 (5.5)	5 (4.1)	11 (7.6)	39 (8.0)α′	59 (8.9)	23 (5.9)	69 (9.7)†	1 (5.2)	3 (6.6)	12 (5.0)	19 (5.7)
*Populus nigra*	67 (7.0)	79 (7.4)	15 (10.6)	9 (8.7)	8 (6.7)	8 (6.1)	62 (12.9)α′	58 (8.7)†	34 (8.6)ω	64 (9.1)	2 (6.6)	5 (8.8)	17 (7.5)	36 (10.8)
*Ambrosia artemisiifolia*	74 (7.8)	90 (8.4)	22(16.1)α′	15 (14.7)	7 (5.8)β′	10 (7.1)	50 (10.4)	88 (13.2)	34 (8.6)β	74 (10.5)	1 (5.2)	5 (8.8)	6 (2.5)	46 (13.8)‡α ′γ
*Artemisia vulgaris*	118 (12.4)	142 (13.2)	25 (18.3)	22 (22.0)	9 (7.7)β′	21 (14.5)	92(19.0)α′α′	139 (20.8)	39 (9.8)βω′	105 (14.9)†	3 (7.9)	8 (16.1)	12 (5.0)	68 (20.3)‡α′δ
*Alternaria alternata*	102 (10.7)	140 (13.0)	12 (8.4)	6 (6.3)	10 (8.6)	14 (9.4)	62 (12.9)	91 (13.6)	56 (14.0)	108 (15.3)	4 (10.3)	7 (14.7)	28 (12.0)	52 (15.6)β

*Note*: ^†^ *p* < 0.05, ^‡^ *p* < 0.001 compared with 2008. ^α^ *p* < 0.05, ^α^′ *p* < 0.001 compared with AR. ^β^ *p* < 0.05, ^β^′ *p* < 0.001 compared with AC.^γ^ *p* < 0.05, ^γ^′ *p* < 0.001 compared with UC. ^ω^ *p* < 0.05, ^ω^′ *p* < 0.001 compared with AR + AC. ^δ^ *p* < 0.05, ^δ^′ *p* < 0.001 compared with AR + UC. ^φ^ *p* < 0.05, ^φ^′ *p* < 0.001 compared with AC + UC. ^ς^ *p* < 0.05, ^ς^′ *p* < 0.001 compared with AR + AC + UC.

Abbreviations: AC, allergic conjunctivitis; AR, allergic rhinitis; AS, asthma; UC, urticaria.

### 2.4 Allergen Profile With Allergic Comorbidity Among Patients With Rhinitis in the Two Cohorts

Table 3 details the allergen profile with allergic comorbidity of rhinitic patients in two cohorts. The respective prevalence rates of allergy to *D. pteronyssinus*, *D. farina*, and *B. tropicalis* in patients with AR and AS (58.2%, 56.4%, 35.7%), AC (56.3%, 54.9%, 34%), UC (54.4%, 52%, 33.6%), or AC + UC (58.5%, 56.2%, 35%) were all higher in the 2018–2019 cohort than those in the 2008–2009 cohort. The percentage of patients with rhinitis and comorbid AC + UC who had *A. vulgaris* allergy was also higher in the 2018–2019 cohort (16.9%) than that in the 2008–2009 cohort (10.8%). By contrast, in the 2008–2009 cohort of rhinitis combined with UC, the prevalence rates of cat (11.5%) and *P. nigra* (4.8%) allergy were higher in comparison with those in the 2018–2019 cohort.

**TABLE 3:** Prevalence (%) of allergen sensitization in allergic comorbidity among patients with rhinitis.

Allergic comorbidity of rhinitis	AS		AC		UC		AS + AC		AS + UC		AC + UC		AS + AC + UC	
Study year	2008–2009, *n* = 171	2018–2019, *n* = 255	2008–2009, *n* = 689	2018–2019, *n* = 723	2008–2009, *n* = 614	2018–2019, *n* = 652	2008–2009, *n* = 80	2018–2019, *n* = 128	2008–2009, *n* = 65	2018–2019, *n* = 127	2008–2009, *n* = 251	2018–2019, *n* = 343	2008–2009, *n* = 49	2018–2019, *n* = 76
*Dermatophagoides pteronyssinus*	78 (45.7)	148 (58.2)‡	281 (40.8)	407 (56.3)‡	231 (37.6)	355 (54.4)‡	39 (48.2)	81 (63.6)†	28 (42.7)	73 (57.1)	97 (38.5)	201 (58.5)‡	25 (50.4)	49 (65.0)
*Dermatophagoides farinae*	73 (42.5)	144 (56.4)‡	268 (38.9)	397 (54.9)‡	220 (35.9)	339 (52.0)‡	37 (46.0)	79 (62.0)†	27 (40.9)	70 (55.0)	93 (36.9)	193 (56.2)‡	24 (48.7)	47 (62.0)
*Blomia tropicalis*	49 (28.9)	97 (35.7)†	176 (25.6)	246 (34.0)‡	128 (20.8)	219 (33.6)‡	24 (30.3)	49 (38.1)	18 (27.5)	46 (36.2)	57 (22.8)	120 (35.0)‡	16 (32.9)	29 (38.5)
Cat dander	22 (12.7)	27 (10.7)	59 (8.5)	59 (8.1)	71 (11.5)	50 (7.6)†	9 (11.7)	16 (12.8)	7 (10.9)	14 (10.7)	28 (11.0)	28 (8.3)	7 (14.7)	10 (13.0)
Dog dander	10 (5.6)	21 (8.4)	35 (5.1)	45 (6.2)	39 (6.3)	42 (6.4)	6 (6.9)	12 (9.3)	5 (7.2)	11 (8.5)	18 (7.0)	26 (7.5)	4 (8.0)	9 (11.2)
Timothy grass	8 (4.9)	17 (6.8)	28 (4.0)	27 (3.7)	24 (3.9)	17 (2.6)^α′^	4 (4.4)	8 (6.3)	3 (5.0)	8 (6.3)	11 (4.2)	15 (4.5)	3 (6.5)	6 (8.2)
*Populus nigra*	9 (5.0)	13 (5.1)	48 (7.0)	39 (5.4)	29 (4.8)	16 (2.5)†^α^	5 (5.9)	8 (6.3)	5 (7.2)	8 (6.3)	18 (7.0)	20 (5.9)	3 (6.5)	4 (5.9)
*Ambrosia artemisiifolia*	7 (4.2)	18 (7.2)	52 (7.5)	56 (7.8)	35 (5.7)	44 (6.8)	5 (6.3)	10 (8.0)	3 (5.0)	12 (9.3)	12 (4.6)	27 (8.0)	4 (7.2)	9 (11.4)
*Artemisia vulgaris*	16 (9.5)	29 (11.5)	96 (14.0)	117 (16.2)	69 (11.3)	82 (12.5)	9 (11.7)	19 (14.8)	6 (7.9)	14 (11.1)	27 (10.8)	58 (16.9)†	6 (12.7)	12 (15.9)
*Alternaria alternata*	16 (9.3)	30 (11.8)	52 (7.6)	42 (5.8)^α′^	43 (7.0)	41 (6.3)^α′^	10 (12.5)	14(11.3)^β^	8 (11.4)	13 (10.5)	19 (7.4)	23 (6.8)	7 (13.3)	10 (13.0)

*Note*: ^†^ *p* < 0.05, ^‡^ *p* < 0.001 compared with 2008. ^α^ *p* < 0.05, ^α′^ *p* < 0.001 compared with AS. ^β^ *p* < 0.05, ^β′^ *p* < 0.001 compared with AC. ^γ^ *p* < 0.05, ^γ′^ *p* < 0.001 compared with UC. ^ω^ *p* < 0.05, ^ω′^ *p* < 0.001 compared with AS + AC. _δ_ *p* < 0.05, ^δ′^ *p* < 0.001 compared with AS + UC. ^φ^ *p* < 0.05, ^φ′^ *p* < 0.001 compared with AC + UC. ^ς^ *p* < 0.05, ^ς′^ *p* < 0.001 compared with AS + AC + UC.

Abbreviations: AC, allergic conjunctivitis; AR, allergic rhinitis; AS, asthma; UC, urticaria.

### 2.5 Association Between IgE Polysensitization and Allergic Multimorbidity in the Two Cohorts

As shown in Table 4, the number of IgE‐reactive allergens increased gradually with the number of comorbid allergic diseases. The number of patients with IgE antibodies against at least one allergen and the number of IgE‐reactive allergens differed significantly among asthma–rhinitis phenotypes between the 2008–2009 and 2018–2019 cohorts. The A+R− and A‐R+ phenotypes showed IgE reactivity to significantly fewer allergens, and these phenotypes exhibited a significantly lower IgE sensitization rate than the A+R+ phenotype in both cohorts (*p* < 0.001). The A+R+ group showed IgE antibodies against significantly more allergens in the 2018–2019 cohort than in the 2008–2009 cohort (*p* < 0.001). The age‐ and sex‐adjusted risk of IgE‐polysensitization was associated with asthma–rhinitis phenotypes and was also significantly higher for the A+R+ groups in the 2008–2009 and 2018–2019 cohorts (adjusted odds ratio [aOR] 1.54, 95% confidence interval [CI] 1.12–1.76 and aOR 1.93, 95% CI 1.53–2.69, respectively; *p* < 0.001) than in all A+R− or A‐R+ groups. Similar change trends were observed in comorbidity phenotypes. The number of patients with IgE reactivity to at least one allergen and the number of IgE‐reactive allergens differed significantly between the comorbidity phenotypes in both cohorts. The groups with at least one comorbidity phenotype showed IgE reactivity to significantly fewer allergens and a significantly lower IgE sensitization rate than the other two comorbidity phenotypes in both cohorts (*p* < 0.001). The three comorbidity phenotypes also showed IgE antibodies to significantly more allergens in 2018–2019 than in 2008–2009 (*p* < 0.001). The age‐ and sex‐adjusted risk of IgE‐polysensitization was also significantly associated with the number of multimorbidities in both cohorts.

**TABLE 4:** Association between the number of IgE‐reactive allergens, asthma–rhinitis phenotypes, and number of comorbidity phenotype.

Dependent variable	Explanatory variable													
	Asthma–rhinitis phenotypes^a^ 2008–2009 Total = 2322			Asthma–rhinitis phenotypes 2018–2019 Total = 2353			Number of multimorbidities^b^ 2008–2009 Total = 2322				Number of multimorbidities 2018–2019 Total = 2353			
	A+R− *N* = 617	A‐R+ *N* = 810	A+R+ *N* = 895	A+R− *N* = 595	A‐R+ *N* = 871	A+R+ *N* = 887	1 *N* = 765	2 *N* = 935	3 *N* = 470	4 *N* = 152	1 *N* = 618	2 *N* = 1033	3 *N* = 459	4 *N* = 243
≥1 positive sIgE antibodies, %	61.8^α,β′^	63.0^β′^	70.6	59.5^α,β′^	67.1^†,β′^	75.1^†^	58.2	68.0^a′^	70.5^a′^	71.7^a′b′^	62.9	70.2^a′^	70.8^a′^	76.6^‡,a′b′c′^
*n*	381	510	632	354	584	666	445	636	331	109	389	725	325	186
Median IgE‐reactive allergens, *n* (25–75 percentiles)	2^α,β′^ (1–2)	2^β′^ (1–2)	2 (1–3)	3^α,β′^ (2–3)	3^†,β′^ (2–3)	3^†^ (2–4)	1 (1–2)	2^a′^ (1–2)	2^a′^ (1–3)	2^a′b′^ (1–3)	2 (2–3)	3^a′^ (2–3)	3^a′^ (2–3)	3^‡,a′b′c′^ (2–3)
IgE‐polysensitization OR (95% CI)^c^	1	1.02 (0.78–1.43)	1.54 (1.12–1.76)Ψ	1	1.50 (1.32–1.79)Ψ	1.93 (1.53–2.69)Ψ	1	1.41 (1.15–1.73)Ψ	2.09 (1.22–3.59)Ψ	2.12 (1.66–2.70)Ψ	1	1.10 (0.79–1.53)	1.65 (1.06–2.28)§	2.20 (1.59–4.64)Ψ

*Note*:^a^ The phenotypes were denoted rhinitis alone (A‐R+), asthma alone (A+R−), and asthma with rhinitis (A+R+).

^b^Comorbidities were asthma, rhinitis, conjunctivitis, and skin disorders. 1: any concomitant allergic disease; 2: any two concomitant allergic diseases; 3: any three concomitant allergic diseases; 4: all concomitant allergic diseases. Skin disorders refer to any of the following: urticaria, atopic dermatitis, and eczema.

^c^Adjusted for age, sex, and region.

†*p* < 0.05, ‡*p* < 0.001 compared with 2008. **^α^** *p* < 0.05, **^α’^** *p* < 0.001 compared with A‐R+. **^β^** *p* < 0.05, **^β’^** *p* < 0.001 compared with A+R+. ^a^ *p* < 0.05, **^a’^** *p* < 0.001 compared with any concomitant allergic disease. ^b^ *p* < 0.05, **^b’^** *p* < 0.001 compared with any two concomitant allergic diseases. ^c^ *p* < 0.05, **^c’^** *p* < 0.001 compared with any three concomitant allergic diseases. Ψ*p* < 0.001. §*p* < 0.05.

Abbreviations: CI, confidence interval; OR, odds ratio; sIgE, specific immunoglobulin E.

### 2.6 Comparison of Environmental Factors Between Monosensitized and Polysensitized Patients in the Two Cohorts

**FIGURE 2:**
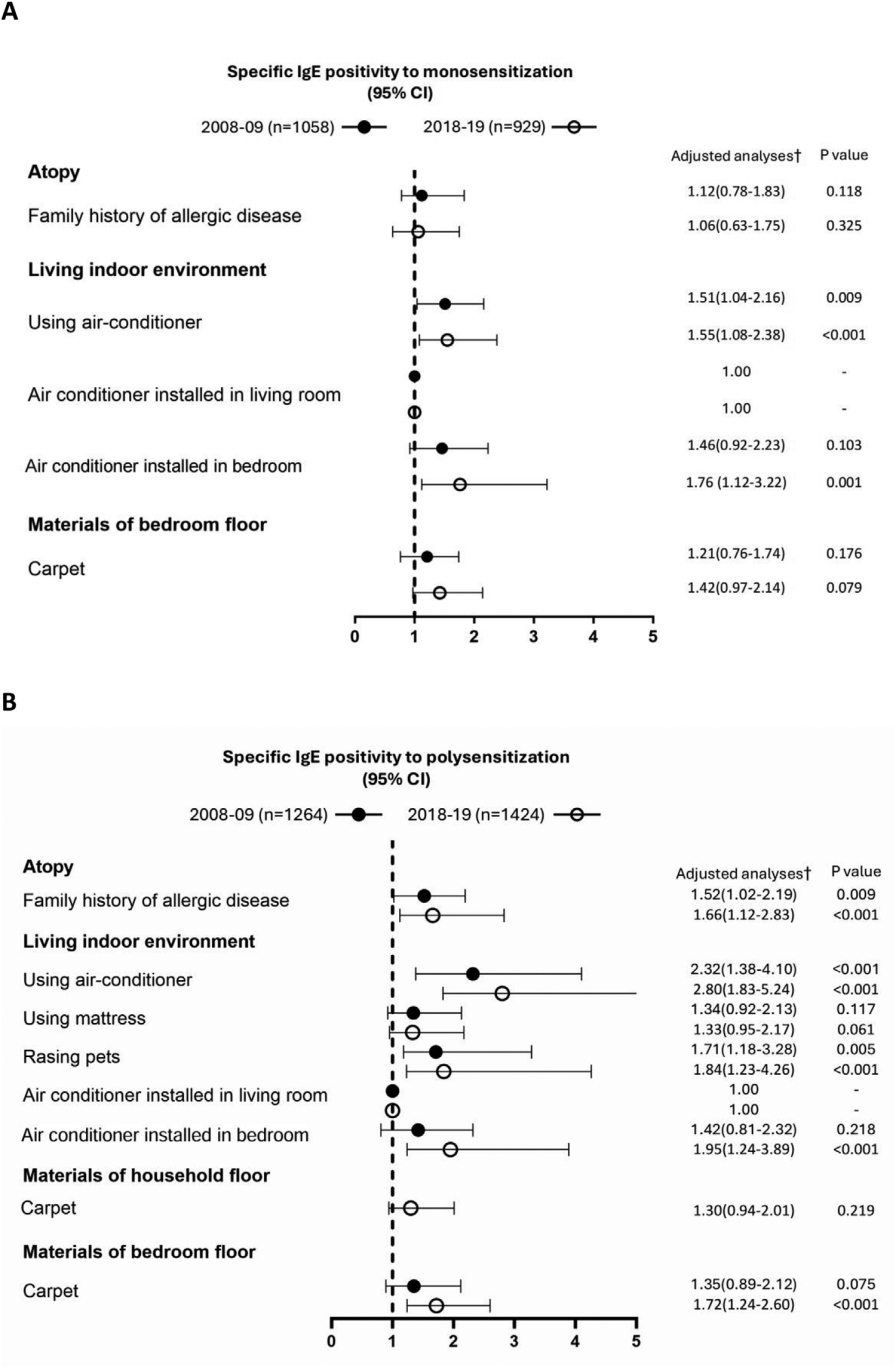
Comparison of risk factors between monosensitized and polysensitized patients using multivariate logistic regression analysis. †Adjusted for age, sex, and region. CI, confidence interval; IgE, immunoglobulin E.

The results of univariate analysis for each of the explanatory variables are presented in Table 5. Figure 2 shows the mutually adjusted OR estimates for all variables selected in the final models. In our multivariate logistic regression model, we found that a family history of allergic disease was a risk factor for polysensitization in both cohorts. Having an air‐conditioner installed in both the living room and bedroom was also a risk factor for monosensitization and polysensitization in the 2018–2019 cohort. Owning a dog or cat was a common risk factor for polysensitization in both cohorts, and having carpet in the bedroom was a risk factor for polysensitization in the 2018–2019 cohort.

**TABLE 5:** Comparison of risk factors between monosensitized and polysensitized patients in univariate logistic regression analysis.

Exposure	Specific IgE positivity to monosensitization (95% CI)				Specific IgE positivity to polysensitization (95% CI)			
	2008–2009, *N* = 1058		2018–2019, *N* = 929		2008–2009, *N* = 1264		2018–2019, *N* = 1424	
	Adjusted analysis^a^	*p* value	Adjusted analysis^a^	*p* value	Adjusted analysis^a^	*p* value	Adjusted analysis^a^	*p* value
Atopy								
Family history of allergic disease	1.42 (1.08–2.04)	0.016	1.38 (1.03–1.95)	0.032	1.55 (1.03–2.46)	0.006	1.89 (1.2–2.71)	< 0.001
Indoor living environment								
Using air‐conditioner	1.67 (1.15–2.56)	0.005	1.53 (1.04–2.21)	0.033	2.02 (1.32–4.46)	< 0.001	3.42 (1.98–5.57)	< 0.001
Using mattress	1.22 (0.76–1.98)	0.359	1.28 (0.87–2.05)	0.142	1.76 (1.44–2.89)	< 0.001	1.84 (1.51–3.18)	< 0.001
Owning pets	0.85 (0.58–1.24)	0.506	1.05 (0.71–1.6)	0.438	1.96 (1.35–3.97)	< 0.001	2.36 (1.65–4.83)	< 0.001
Air‐conditioner installed in living room	1	—	1	—	1	—	1	—
Air‐conditioner installed in bedroom	1.62 (1.05–2.18)	0.012	1.9 (1.16–3.67)	0.004	1.73 (1.08–2.55)	0.018	2.19 (1.33–4.25)	< 0.001
Household floor material								
Wood	1.02 (0.73–1.41)	0.752	0.82 (0.27–1.73)	0.353	0.89 (0.32–1.81)	0.663	1.12 (0.79–1.59)	0.304
Carpet	1.13 (0.53–1.96)	0.553	1.05 (0.66–1.64)	0.747	1.45 (0.83–2.41)	0.072	2.12 (1.24–2.97)	0.028
Bedroom floor material								
Wood	0.88 (0.47–1.45)	0.582	1.02 (0.65–1.56)	0.861	0.83 (0.42–1.75)	0.226	0.86 (0.39–1.94)	0.286
Carpet	1.55 (1.09–2.18)	0.008	1.62 (1.21–2.35)	< 0.001	1.49 (1.05–2.13)	0.018	2.33 (1.94–2.87)	< 0.001
Dietary habits (two or more times per week)								
Red meat	0.76 (0.49–1.35)	0.298	0.86 (0.58–1.84)	0.378	0.68 (0.31–1.33)	0.48	0.72 (0.34–1.51)	0.548
Fried fish	0.83 (0.62–1.49)	0.186	0.94 (0.67–2.03)	0.651	0.77 (0.48–1.57)	0.34	1.15 (0.85–1.74)	0.414
Fresh fruits	1.05 (0.72–1.63)	0.635	1.25 (0.83–2.95)	0.118	0.88 (0.43–1.86)	0.675	1.08 (0.76–1.97)	0.561
Raw vegetables	1.28 (0.81–2.05)	0.109	1.36 (0.88–2.29)	0.078	1.06 (0.78–2.03)	0.221	1.26 (0.79–1.98)	0.638
Fruit juices	1.04 (0.73–2.93)	0.423	1.31 (0.98–2.54)	0.083	1.12 (0.65–1.73)	0.439	1.1 (0.63–1.68)	0.436
Soft drinks	1.08 (0.75–1.68)	0.434	1.24 (0.81–2.89)	0.095	1.22 (0.75–1.92)	0.098	1.18 (0.69–1.74)	0.167

Abbreviations: CI, confidence interval; IgE, immunoglobulin E.

^a^ Adjusted for age, sex, and region.

## 3 Discussion

The present study findings showed that allergic symptoms and allergen sensitization were significantly greater among mono‐ and polysensitive patients in the 2018–2019 cohort, as compared with the 2008–2009 cohort. Furthermore, the prevalence of comorbidity in asthma, AR, AC, and eczema was significantly increased in the 2018–2019 cohort. Environmental factors including air‐conditioner use, pet ownership, and carpet exposure were associated with polysensitization. These findings indicate that during the 10‐year study period in China, the phenomenon of comorbid allergic diseases in China became more prevalent, with increased allergen sensitivity.

We found a similar prevalence of multiple comorbidities in both sexes for asthma, AR, AC, and eczema, similar to previous studies [8, 9]. This suggests that sex has a lesser influence on the coexistence of multiple allergic diseases. Asthma often coexists with other allergic diseases rather than occurring alone. A Korean and Chinese birth cohort previously reported that more than half of children with asthma symptoms had coexisting rhinitis and/or eczema [10, 11]; however, precise adult data were lacking. In our study, the comorbidity rates of eczema, AR, and asthma were significantly increased in the 2018–2019 cohort, suggesting that there may be common triggers or mutually aggravating effects on the pathophysiological mechanisms of these diseases. These findings also highlight the importance of systematic comorbidity evaluation in adult asthma [12]. The commonly used term “atopic march” may reflect the natural history and progression of eczema, asthma, and AR at a cross‐sectional, population‐based level rather than the trajectory of allergic disease development in individuals [13, 14]. As shown in our forest plot, the use of air‐conditioners may be the primary factor. The second factor is pet‐raising, the third is the use of carpets, followed by a family history of atopy and the use of mattresses. These findings suggest that both genetic and environmental exposure might significantly increase the risk of allergic comorbidities and contribute to the differences in prevalence over the 10‐year study period, similar to other studies [8, 15, 16].

Environmental factors have been implicated simultaneously in the individuals. These factors generally do not exist in isolation; instead, they interact synergistically and jointly exert effects on the human health [17]. On the one hand, air flow driven by air‐conditioner has a significant impact on the indoor environment. Previous research indicated that a high microbial count was present in the air outlet, filter net, cooling fin, and water sink of air‐conditioner [18]. Dust mites were generally detected in the dust samples from filter net, and there was close relationship between cleaning frequency of filter net with mite breeding rate [19]. There were also data suggesting that condensation on the water sink enabled the accumulation of hidden moisture and the growth of fungi, particularly in air‐conditioner that had been in use for ≥ 6 years or were used frequently during the summer [20]. Both exposure to dust mite and mold taxa were found to be associated with the development of upper respiratory tract (nasal and throat) symptoms and asthma (wheeze and cough) symptoms. These could explain why the risk of polysensitization increased when using an air‐conditioner in the bedroom. On the other hand, the presence of carpets in homes was a strong determinant for cat allergen, mold glucan, and dust mites loadings [21]. This could be a reason for the carpet flooring, which was strongly associated with polysensitization.

Can f1 and Fel d1 allergens are mainly found in dog and cat dander and are present in small particles in the air and carpets in homes with dogs and cats [22]. In the study, we considered pet ownership as an integral variable and found that pet keeping (no matter cats or dogs) was strongly associated with the presence of multiple allergic disorders (asthma, rhinitis, and eczema), which accords with the earlier observation [23]. There was also some indication suggesting that ownership of furry animals in early life may offer some protection against asthma in the absence of animal‐specific sensitization [24]. However, within our cohort (predominantly adults), these potential protective mechanisms might not have been fully actualized, and cross‐reactivity between dog and cat allergens may also be taken into consideration.

The prevalence of airborne allergen‐related IgE sensitization was higher in the 2018–2019 cohort than that in the 2008–2009 cohort, which can explain the notable increase in the number of IgE‐reactive allergens in the former cohort. This finding further supports the close relationship between the coexistence of allergic diseases and IgE sensitization [25, 26]. Similarly, in both cohorts, the risk of IgE reactivity was highest in the asthma–rhinitis multimorbid phenotype, as compared with the isolated asthma or rhinitis phenotypes; this is also consistent with previous reports [27].

To better understand the relationship between IgE polysensitization and different allergic multimorbidities, we extracted patient data on seven allergic diseases (AR, AC, UC, AR + AC, AR + UC, AC + UC, and AR + AC + UC) for analysis. Our results showed that the positivity rate of sensitization in patients with airway allergic multimorbidities combined with UC/eczema was higher than other entities.

In addition, when airway allergic diseases were complicated with UC/eczema, the mite IgE level was higher than that in only airway allergic diseases. Therefore, recent studies indicated that filaggrin mutations and skin barrier defects lead to enhanced skin irritability to non‐specific stimuli and epicutaneous sensitization, including house dust mite (HDM). In fact, most eczema patients are sensitized to HDM allergens, which are important factors in exacerbating symptoms [28, 29]. Some studies showed that teen group were more frequently diagnosed with allergic multimorbidities than adults [30, 31]. While this was a cross‐sectional study, we were not able to discern whether asthma or rhinitis tended to become a single condition as children grow older.

There were few multicenter surveys focusing on asthma‐rhinitis multimorbidity characteristics in Chinese Mainland prior to this study. The main strength of this study lies in the fact that allergic outcomes were determined using strict epidemiologic definitions, which ensures reliability and representativeness of the findings. The selection of identical designs in CARRAD‐I and CARRAD‐II was motivated by the necessity for the aim of comprehensively uncovering the complexity and intrinsic relationships of allergy‐related issues. However, this study also has several limitations. First, although our data support the polydysplastic nature of the investigated allergic diseases, our data support the allergic multimorbidity nature of these conditions, but 2008–2009 cohort and 2018–2019 cohort do not represent a follow‐up of the same individuals, so we lack longitudinal data that would allow us to further explore the progression and mechanisms of atopic comorbidities. Second, as a cross‐sectional study, it is not possible to establish causal relationships. Further, despite controlling for multiple confounding factors, self‐selection bias and information bias (e.g., behavioral bias and recall bias) may have influenced the results. The access to healthcare services and public awareness of allergic disease over the 10‐year period may have influenced the observed results. Using air‐conditioner and mattress were reported retrospectively, thus the logistic regressions are also vulnerable to recall bias, although their impact would be minimal, limiting the generalizability of the findings.

In summary, the study demonstrated that over time, the co‐occurrence of allergic diseases has become more prevalent in the Chinese population, with an increase in allergen sensitivity. Allergic diseases should be considered as systemic diseases with different manifestations at different sites. More and more evidence suggest that “one allergy caused by one exposure through one pathway.” The concept of “one allergy” indicates that environmental control measures for asthma can offer a reference for the prevention of AR, AC, and UC simultaneously [32]. Our findings will provide crucial epidemiologic evidence for experts in public health and allergy medicine toward the development of treatment strategies to systematically address the coexistence of multiple allergic conditions. Future research should further investigate how environmental and lifestyle changes affect the co‐occurrence of allergic diseases to better prevent and manage these conditions.

## 4 Materials and Methods

### 4.1 Study Design

We redesigned the dataset of the CARRAD two stage real‐world studies and maintain methodological consistency [2]. After stratified sampling, a subsample of 2322 and 2353 patients, respectively, who attended outpatient clinics in respirology, allergology, otorhinolaryngology, or pediatrics were enrolled. The inclusion criteria were as follows: patients aged 5–65 years, those diagnosed with rhinitis and/or asthma based on a physician's diagnosis, and those with data regarding an atopic history and IgE levels. Patients who refused to participate in the surveys or who were unable to understand or complete the questionnaires were excluded. A rigorous protocol, questionnaire, allergen testing set, and operating procedures were used at all centers of CARRAD, and the interviewers were trained before study. All participants were asked to sign a consent form to provide blood for measurement of serum IgE levels.

### 4.2 Questionnaire

The questionnaire was adapted from the version of standardized questionnaire utilized in the second phase of the International Study of Asthma and Allergies in Childhood (ISAAC‐II) project [33]. The classification of allergic multimorbidity was based on standardized self‐reported questions that have been validated in ISAAC‐II. This version is identical to the one employed in our research centered on CARRAD‐I, with certain modifications made in accordance with the actual circumstances in China (Table S1). The questionnaire was administered in person by physicians or research nurses, encompassing inquiries regarding the subsequent baseline demographic characteristics: family history of atopy; nasal manifestations, including rhinorrhea, itching, sneezing, and blockage, as well as pulmonary symptoms, such as wheezing, coughing, and chest tightness; cutaneous and ocular symptoms, like eczema and burning or itchy eyes; smoking behaviors; environmental exposure elements; pet ownership; and eating habits. Questions concerning the influence of allergic symptoms on daily activities, work or school, nocturnal sleep, and the utilization of medications for symptom control were also incorporated. According to the guideline AR and its Impact on Asthma (ARIA), ever asthma was defined as an affirmative answer to either “Have you ever had attacks of breathlessness at rest with wheezing?” or “Have you ever had asthma attacks?” or ever having been recruited as an asthma case. Asthma was defined as having a history of recurrent dyspnea, wheezing, or coughing episodes; positive airway reversibility testing (forced expiratory volume in the first second [FEV1]) increased ≥ 12% and 200 mL after inhalation of 400 mg salbutamol; or positive airway responsiveness testing (FEV1 decreased ≥ 20% with administration of a ≤ 7.8 µg cumulative dose of histamine) [34]. Ever AR was defined as an affirmative answer to “Have you ever had rhinitis?” or “Have you ever had hay fever?” or being recruited as a rhinitis case. Rhinitis was defined as having symptoms of sneezing or a runny, itchy, or blocked nose in the absence of a cold or influenza [35]. Urticaria was defined as a condition characterized by the development of wheals (hives) and angioedema [36]. Inflammation of the conjunctiva was characterized as pruritus and watery, red eyes with positive sIgE to whole allergens, or their purified molecular components [37]. Allergic multimorbidity at each time point was defined as the coexistence of at least two of the following diseases in one participant: asthma, AR, eczema/urticaria, and conjunctivitis.

### 4.3 Serum IgE Measurement

A peripheral blood sample of 10 mL was taken from each individual; coagulated at room temperature, centrifuged, and stored at 4°C; and sent to the central laboratory at Guangzhou Medical University every month. The sIgE levels against *Dermatophagoides pteronyssinus*, *D. farinae*, *Blomia tropicalis*, cat dander, dog dander, timothy grass, *Populus nigra*, *Ambrosia artemisiifolia*, *Artemisia vulgaris*, and *Alternaria alternata* were measured using the ADVIA Centaur immunoassay system (Siemens AG, Erlangen, Germany). The sIgE cutoff value was set at 0.35 IU/mL, and a positive response was defined as an sIgE level ≥ 0.35 IU/L.

### 4.4 Statistical Analysis

The baseline characteristics and prevalence in allergic comorbidities between the two cohorts were compared using chi‐square tests. A two‐sample *t*‐test was used to analyze normally distributed variables, and the Mann–Whitney *U*‐test was used to analyze non‐normally distributed variables. Prevalence rates of aeroallergen sIgE positivity among patients with monosensitization and polysensitization were compared using chi‐square tests. Logistic regression analyses were performed to test for any association between explanatory variables and clinical outcomes (number of IgE‐reactive allergens and number of comorbidity phenotypes). Univariable logistic regression analyses were performed to study the associations between family atopic history and environmental factors as well as dietary habits and monosensitization or polysensitization. Each of the explanatory variables was included in a model together with sex, age, and region variable. The explanatory variables that were associated with the dependent variable (overall *p* value for the explanatory variable < 0.05) were considered potential risks or protective factors of monosensitization or polysensitization and were introduced into the stepwise multivariable model. The criteria for entering and removing a variable were probability of *F* to enter 0.05 and probability of *F* to remove 0.10. All variables for inclusion were carefully chosen, given the number of events available, to ensure parsimony of the final model. The results are presented as aORs with 95% CIs. All data were analyzed using IBM SPSS version 21.0 (IBM Corp., Armonk, NY, USA) and R software (version 4.3.1). A two‐tailed *p* value <0.05 was considered statistically significant.

## Author Contributions

Jing Li, Nanshan Zhong, Jianhong Wang, Guihua Song, Hua Xie, Rongfei Zhu, Yong He, Jun Tang, Junge Wang, Jinghua Yang, Lili Zhi, Lin Wu, Yan Jiang, Xiaoqin Zhou, Dongming Huang, Ning Wang, Rui Xu, Yuan Gao, Zhimin Chen, Jinling Liu, Xiaoli Han, Guolin Tan, Jinzhun Wu, Deyu Zhao, Jianjun Chen, Xiwei Zhang, Yuemei Sun, Yi Jiang, Weitian Zhang, Qianhui Qiu, Chuanhe Liu, Jie Yin, Guodong Hao, Huabin Li, Yong Sheng Xu, Shaohua Chen, Shi Chen, Juan Meng, Dan Zeng, Wei Tang, and Chuangli Hao had the idea for and designed the study. Jing Li supervised the study and did the writing – review. Wanjun Wang did the data curation and wrote the original draft. All authors contributed to acquisition, analysis, or interpretation of data. All authors revised the report and approved the final version before submission. All authors contributed equally to the study.

## Funding

This study was supported by the National Natural Science Foundation of China (82161138020) and Major Project of Guangzhou National Laboratory (Grant No. GZNL2024A02002). Noncommunicable Chronic Diseases‐National Science and Technology Major Project (2024ZD0529900)

## Ethics Statement

This research was carried out in accordance with the Declaration of Helsinki (as revised in 2013). Written informed consent was obtained from all participants. The study protocol was approved by the Ethics Committee of the First Affiliated Hospital of Guangzhou Medical University (ES‐20180302).

## Conflicts of Interest

The authors declare no conflicts of interest.

## Supporting information




**Supporting Information**: mco270762‐sup‐0001‐SuppMat.docx

## Data Availability

The raw data that support the findings of this study are available from the corresponding author upon reasonable request.
