# Supplementary material for: Trends in Asthma‐Rhinitis Allergic Multimorbidity and Polysensitization in China: The CARRAD Study
Source: MedComm (2020). 2026 Jun 4;7(6):e70762. doi: 10.1002/mco2.70762 (PMC13238639; doi:10.1002/mco2.70762)
Supplement: Supplementary file 1 — Supporting Information: mco270762‐sup‐0001‐SuppMat.docx [file MCO2-7-e70762-s001.docx]

**Trends in asthma-rhinitis allergic multimorbidity and poly-sensitization in China: the CARRAD study.**

Wanjun Wang^1#^; Jianhong Wang^2#^; Guihua Song^3#^; Hua Xie^4#^; Rongfei Zhu^5#^; Yong He^6#^; Jun Tang^7#^; Junge Wang^8#^; Jinghua Yang^9#^; Lili Zhi^10#^; Lin Wu^11#^; Yan Jiang^12#^; Xiaoqin Zhou^13#^; Dongming Huang^14#^; Ning Wang^15#^; Rui Xu^16#^; Yuan Gao^17#^; Zhimin Chen^18#^; Xiaoli Han^19#^; Guolin Tan^20#^; Jinzhun Wu^21#^; Deyu Zhao^22#^; Jianjun Chen^23#^; Xiwei Zhang^24#^; Yuemei Sun^25#^; Yi Jiang^26#^; Weitian Zhang^27#^; Qianhui Qiu^28#^; Chuanhe Liu^29#^; Jie Yin^30#^; Guodong Hao^31#^; Huabin Li^32#^; Yongsheng Xu^33#^; Shaohua Chen^34#^; Shi Chen^35#^; Juan Meng^36#^; Dan Zeng^37#^; Wei Tang^38#^; Chuangli Hao^39#^; Nanshan Zhong^1*^; Jing Li^1*^; on behalf of the China Alliance of Research on Respiratory Allergic Disease (CARRAD)^*^

1 Department of Allergy and Clinical Immunology, National Clinical Research Center for Respiratory Disease, State Key Laboratory of Respiratory Disease, Guangzhou Institute of Respiratory Health, The First Affiliated Hospital of Guangzhou Medical University, Guangzhou, China

2The First People's Hospital of Yibin, Yibin, Sichuan, China

3The First Affiliated Hospital of Henan University of Traditional Chinese Medicine, Zhengzhou, Henan, China

4General Hospital of Northern Theater Command, Shenyang, China

5Tongji Hospital, Tongji Medical College, Huazhong University of Science & Technology, Wuhan, China

6The Affiliated Hospital of Medical School, Ningbo University, Ningbo, Zhejiang, China

7Foshan First People's Hospital, Foshan, China

8Beijing Hospital of Traditional Chinese Medicine, Beijing, China

9Guangdong Provincial Hospital of Chinese Medicine, Guangzhou, China

10The First Affiliated Hospital of Shandong First Medical University, Shandong Institute of Respiratory Diseases, Taian, China

11Hangzhou Hospital of Traditional Chinese Medicine, Hangzhou, China

12The Affiliated Hospital of Qingdao University, Qingdao, China

13Hubei Province Maternal and Child Health Hospital, Wuhan, Hubei, China

14Boai Hospital of Zhongshan City, Zhongshan, China

15Xi'an Children's Hospital, Xi'an, Shaanxi, China

16The First Affiliated Hospital of Sun Yat-sen University, Guangzhou, China

17The First Affiliated Hospital of Zhengzhou University, Zhengzhou, China

18Children's Hospital of Zhejiang University School of Medicine, National Clinical Research Center for Child Health, Hangzhou, China

19Hebei General Hospital, Shijiazhuang, Hebei, China

20 Third Xiangya Hospital of Central South University, Changsha, China

21 The Women and Children's Hospital Affiliated to Xiamen University, Xiamen, China

22 Children's Hospital of Nanjing Medical University, Nanjing, China

23 Union Hospital of Tongji Medical College, Wuhan, China

24 The Second Affiliated Hospital and Yuying Children's Hospital of Wenzhou Medical University, Wenzhou, China

25 Yu Huang Ding Hospital, Yantai, China

26 The First Hospital of Shanxi Medical University, Taiyuan, China

27 Shanghai Jiao Tong University Affiliated Sixth People's Hospital, Shanghai, China

28 Zhujiang Hospital of Southern Medical University, Guangzhou, China

29 Children's Hospital Capital Institute of Pediatrics, Beijing, China

30 Chengdu First People's Hospital, Chengdu, China

31 Tangshan Gongren Hospital, Tangshan, Hebei, China

32 ENT Institute and Department of Otorhinolaryngology, Eye & ENT Hospital, Fudan University, Shanghai, China

33 Children's Hospital of Tianjin University, Tianjin, China

34 Guangdong Provincial People's Hospital, Guangzhou, China

35 Hainan Provincial People's Hospital, Haikou, China

36 West China Hospital of Sichuan University, Chengdu, China

37 Chongqing General Hospital, University of Chinese Academy of Sciences, Chongqing, China

38 Ruijin Hospital of Shanghai Jiaotong University, Shanghai, China

39 Children's Hospital of Soochow University, Suzhou, China

**Correspondence**

Jing Li and Nanshan Zhong, Department of Allergy and Clinical Immunology, National Clinical Research Center for Respiratory Disease, State Key Laboratory of Respiratory Disease, Guangzhou Institute of Respiratory Health, The First Affiliated Hospital of Guangzhou Medical University, Guangzhou 510120, China. Email: lijing@gird.cn; nanshan@vip.163.com

**Screening Questionnaire**

**We are seeking your collaboration for a "Multicenter Clinical Questionnaire on Allergens in the Chinese Population". This questionnaire is outlined as below. Please be assured that the data will be exclusively utilized for scientific analysis, and your personal information will be maintained with the utmost confidentiality. We sincerely appreciate your support and cooperation!**

1. Name:*

| Clinic number: |
| --- |
| Contact phone number: |
| 2. Date ofbirth:* |

3. Gender: [Single-choice question]* ○Male ○Female

4. Ethnicity [Optional]*

○ Han Chinese ○ Uyghur ○ Tibetan ○ Hui ○ Others

5. Place of residence [Optional]*

○Native ○Immigrant, Years of Residence

6. Are you a smoker [Multiple-choice question]*

○ Currently still smoking ○ Never smoked ○ Quit smoking, how many years quit

7. How many cigarettes, if any, do you smoke per day? [Single-choice question] *

○ Less than 10 ○ 10 to 20 ○ More than 20

8. At what age did you start smoking? [Fill in the blank] *


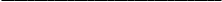


9. How many people smoke in your household? [Fill in the blank] *

___________________________________

10. Does anyone in your family suffer from allergic diseases? (If not, please skip to Question 17)* ○Yes ○No

11. Has your father ever suffered from any of the following? [Multiple-choice question] *

□ Allergic rhinitis □ Bronchial asthma □ Eye allergies □ Food allergies

□ Allergic dermatitis □ No □ Other, please specify

12. Has your mother ever suffered from any of the following? [Multiple-choice question] *

□ Allergic rhinitis □ Bronchial asthma □ Eye allergies □ Food allergies

□ Allergic dermatitis □ No □ Other, please specify

13. Do you have an older or younger brother? (If not, please skip to Question 15) * ○Yes ○No

14. If yes, has the elder or younger brother ever suffered from any of the following diseases? [Multiple-choice question] *

□ Allergic rhinitis □ Bronchial asthma □ Eye allergies □ Food allergies

□ Allergic dermatitis □ No □ Other, please specify

15. Do you have an older or younger sister? (If not, please skip to Question 17) [Single-choice question]* ○Yes ○No

16. If yes, has the elder or younger sister ever suffered from any of the following diseases? [Multiple-choice question] *

□ Allergic rhinitis □ Bronchial asthma □ Eye allergies □ Food allergies

□ Allergic dermatitis □ No □ Other, please specify

17. Have any of your other relatives ever suffered from any of the following diseases? [Multiple-choice question] *

□ Allergic rhinitis □ Bronchial asthma □ Eye allergies □ Food allergies

□ Allergic dermatitis □ No □ Other, please specify

18. Do you have or have you ever had nose allergies (including chilblain fever)? [Single-choice question] * ○Yes ○No

19. Over the past 12 months, have you ever had a sneeze, runny nose, or stuffy nose when you did not catch a cold or flu? [Multiple-choice question] *

○Yes ○No

20. Overall the past 12 months, have you had any contact with grass, trees or flowers? (If no, please skip to Question 22) [Single-choice question]*

○Yes ○No

21. If yes, have you experienced symptoms of itching, nasal congestion or sneezing? [Multiple-choice question] * ○Yes ○No

22. Over the past 12 months, have you had any contact with animals, such as horses, dogs, cats or voles with fur? (If no, please skip to Question 24) [Single-choice question] *

○Yes ○No

23. If yes, have you felt an itchy or stuffy nose or have you sneezed? [Single-choice question] * ○Yes ○No

24. In which months did you have the above nasal symptoms (sneezing, runny nose, itchy nose or stuffy nose)? [Multiple-choice question] *

January □ February □ March □ April □ May □ June □ July

□August □September □October □November □December □None

25. Do you have or have you ever had seasonal nasal allergies (chilblain fever)? [Single-choice question] * ○Yes ○No

26. Do you have or have you ever had allergic rhinitis? [Multiple-choice question] * ○Yes ○No

27. Over the past 12 months, how has the above nasal discomfort affected your daily life? [Multiple-choice question] * ○No impact ○Slight impact ○Moderate impact ○Serious impact

28. What are the episodes of nasal discomfort described above: [Single-choice question]* ○Seasonal ○Annual ○Irregular

29. Do you have or have you ever had eye allergies (itchy, watery or red eyes)? (If no, please skip to Question 31) [Single-choice question]*

○Yes ○No

30. If yes, do your eye symptoms coincide with symptoms of nasal discomfort? [Multiple-choice question] * ○Yes ○No

31. Over the past 12 months, did you have a gritty feeling, redness, watering or itching in your eyes when you did not have a cold? [Single-choice question] *

○Yes ○No

32. Over the past 12 months, have you had any contact with animals with fur (e.g., cats, dogs, horses, rats, etc.)? (If no, please skip to Question 34) [Single-choice question]*

○Yes ○No

33. If yes, do your eyes have a gritty feeling, redness, watering or itching? [Single-choice question] * ○Yes ○No

34. Over the past 12 months, have you had any contact with grass, trees or flowers? (If no, please skip to Question 36) [Single-choice question]*

○Yes ○No

35. If yes, do you have a gritty feeling, redness, tearing or itching in your eyes? [Single-choice question] *

○Yes ○No

36. In which month did the above symptoms occur in your eyes? [Multiple-choice question] * January □ February □ March □ April □ May □ June □ July

□August □September □October □November □December □None

37. Over the past 12 months, have you had symptoms of wheezing or croup? [Multiple-choice question] * ○Yes ○No

38. Over the past 12 months, have you ever woken up at night from the tightness in your chest or the tightness of breath? [Single-choice question] *

○Yes ○No

39. Over the past 12 months, have you ever woken up at night with a cough? [Single-choice question] * ○Yes ○No

40. Have you had any asthma attacks over the past 12 months? [Multiple-choice question] * ○Yes ○No

41. Over the past 12 months, have you used nebulized inhalation medications to facilitate your breath? [Single-choice question] *

○Yes ○No

42. Are you currently experiencing any medications (including inhaled or oral medications) for the treatment of your asthma?

[Multiple-choice question] * ○Yes ○No

43. Over the past 12 months, have you touched grass, trees or flowers? (If no, please skip to Question 45) [Single-choice question]*

○Yes ○No

44. If yes, have you experienced any symptoms of breathlessness or wheezing? [Multiple-choice question] * ○Yes ○No

45. Over the past 12 months, have you had any contact with animals such as horses, dogs, cats or voles? (If no, please skip to Question 47) [Single-choice question] *

○Yes ○No

46. If yes, do you experience any symptoms of breathlessness or wheezing? [Multiple-choice question] * ○Yes ○No

47. In the past 12 months, have you experienced symptoms of breathlessness or wheezing when you exercise or after exercising? [Multiple-choice question] *

○Yes ○No

48. Have you ever suffered from hives or other skin allergies? (If no, please skip to Question 50) [Single-choice question] * ○Yes ○No

49. Have you suffered from a recurring itchy rash over the past 6 months? [Single-choice question] * ○Yes ○No

50. Does anyone in your family or any of your relatives suffer from allergies? (If no, please skip to Question 53) [Single-choice question] *

○Yes ○No

51. What about the allergies run in your family [Multiple-choice question]*

□ Parent □ Sibling □ Child

52. What is the type of allergy? [Fill in the blank] *


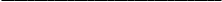


53. How old was your mother when you were born? [Fill in the blank] *


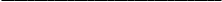


54. How many years of education do you have? [Fill in the blank] *


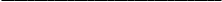


55. How many brothers and sisters do you have? [Fill in the blank] *


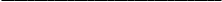


56. How many brothers and sisters do you have? [Fill in the blank] *

___________________________________

57. How often do you eat meat on average? [Single-choice question] *

○No ○Less than once a week ○Monthly to twice a week ○Wednesday to six times a week ○More than once a day

58. How often do you eat fish on average? [Multiple-choice question] *

○No ○Less than once a week ○Monthly to twice a week ○Wednesday to six times a week ○More than once a day

59. How often do you eat fruit on average? [Single-choice question] *

○No ○Less than once a week ○Monthly to twice a week ○Wednesday to six times a week ○More than once a day

60. How often do you eat raw green vegetables on average? [Single-choice question] *

○No ○Less than once a week ○Monthly to twice a week ○Wednesday to six times a week ○More than once a day

61. How often do you eat cooked green vegetables on average? [Single-choice question] *

○No ○Less than once a week ○Monthly to twice a week ○Wednesday to six times a week ○More than once a day

62. How often do you drink fruit juice on average? [Single-choice question] *

○No ○Less than once a week ○Monthly to twice a week ○Wednesday to six times a week ○More than once a day

63. How often do you drink soda on average? [Multiple-choice question] *

○No ○Less than once a week ○Monthly to twice a week ○Wednesday to six times a week ○More than once a day

64. Do you consume processed cooking oils (e.g., peanut, canola or soybean oil) in your household [Single-choice question]* ○Yes ○No

65. When was the house you are living in built? [Fill in the blank] *


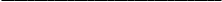


66. What does the house you are living in look like? [Single-choice question] *

○Multi-story (less than nine stories) ○High-rise (more than nine stories) ○Bungalows

○One floor? (less than two households, two to four households, more than five households) ○ Other

67. Where do you live? [Single-choice question] * ○Cities ○Rural areas ○Towns ○Suburbs

68. How many people live in your house? [Fill in the blank] *


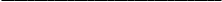


69. How many rooms are there in your house? (Excluding kitchen, bathroom, toilet and laundry) [Fill in the blank] *

___________________________________

70. Do you have air conditioning in your home? (If not, please skip to question 72) [Single-choice question]* ○Yes ○No

71. In which house is the air conditioner installed? [Multiple-choice question] *

□ Living room □ Your bedroom □ Both

72. What is the living room floor in your home? [Multiple-choice question]*

□ Tile or concrete □ Wooden flooring □ Laminate flooring □ Plastic flooring □ Carpeting

73. What is your bedroom floor? [Multiple-choice question]*

□ Tile or concrete □ Wooden flooring □ Laminate flooring □ Plastic flooring □ Carpeting

74. What kind of pillow are you using? [Multiple-choice question] *

□ Cotton □ Sponge □ Synthetic material □ Feather □ Plant-based (herbal)

75. Are you using a mattress? [Single-choice question] * ○Yes ○No

76. What kind of quilt are you using? [Multiple-choice question] *

□ Quilt □ Synthetic material □ Feather □ Blanket □ Other

77. Which of the following devices are you using to heat your home? [Multiple-choice question] *

□ Piped gas, bottled LPG □ Air conditioner □ Other

78. Which of the following devices are you using to boil hot water? [Multiple-choice question] *

□ Electric water heater □ Gas water heater □ Electric kettle □ Gas kettle □ Kerosene kettle □ Other

79. Which of the following devices are you using for cooking? [Multiple-choice question] *

□ Electricity □ Gas or LPG □ Coal or firewood □ Kerosene □ Other

80. Do you own a cat? (If not, please skip to Question 82) [Single-choice question] * ○Yes ○No

81. If yes, for how many years have you kept the cat? [Fill in the blank] *


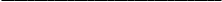


82. Do you own a dog? (If not, please skip to Question 84) [Single-choice question]* ○Yes ○No

83. If yes, for how many years have you kept the dog? [Fill in the blank] *


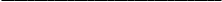


84. Do you own birds? (If not, please skip to Question 86) [Single-choice question]* ○Yes ○No

85. If yes, for how many years have you kept the birds? [Fill in the blank] *


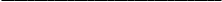


86. Do you have any other pets? (If not, please skip to Question 88) [Single-choice question]* ○Yes ○No

87. If yes, for how many years have you kept the pets? [Fill in the blank] *

___________________________________

88. Do you allow pets in your house? [Multiple-choice question] * ○Yes ○No

89. Do you allow pets in your bedroom? [Multiple-choice question] *

○Yes ○No

90. Have you ever owned a pet before? [Single-choice question] * ○Yes ○No

91. How old were you when you kept a pet? [Single-choice question] *

○ How long did you raise when you were 1 year old?

○ How long did you raise when you were 1 to 4 years old?

○ How long did you raise when you were 5 to 15 years old?
